# Molecular characterization of constitutive heterochromatin in three species of *Trypoxylon* (Hymenoptera: Crabronidae: Trypoxylini) by CMA3/DAPI staining

**DOI:** 10.3897/compcytogen.v5i2.961

**Published:** 2011-07-01

**Authors:** Rodolpho Santos Telles Menezes, Antonio Freire Carvalho, Janisete Gomes Silva, Marco Antonio Costa

**Affiliations:** *Departamento de Ciências Biológicas, Universidade Estadual de Santa Cruz. Rod Ilhéus/Itabuna Km 16, 45650-000 Ilhéus, Bahia, Brazil*; *Departamento de Ciências Biológicas, Universidade Estadual de Santa Cruz. Rod Ilhéus/Itabuna Km 16, 45650-000 Ilhéus, Bahia, Brazil*; *Departamento de Ciências Biológicas, Universidade Estadual de Santa Cruz. Rod Ilhéus/Itabuna Km 16, 45650-000 Ilhéus, Bahia, Brazil*; *Departamento de Ciências Biológicas, Universidade Estadual de Santa Cruz. Rod Ilhéus/Itabuna Km 16, 45650-000 Ilhéus, Bahia, Brazil*

**Keywords:** Comparative cytogenetics, heterochromatin, CMA3/DAPI

## Abstract

Previous cytogenetic analyses in *Trypoxylon* Latreille, 1796 have been basically restricted to C-banding. In the present study, base-specific CMA3 and DAPI fluorochrome staining were used to characterize the constitutive heterochromatin in three *Trypoxylon* species*.* The heterochromatin was GC-rich in all the species studied; however, in *Trypoxylon nitidum* F. Smith, 1856the molecular composition of the heterochromatinwasdifferent among chromosome pairs. Conversely, the euchromatin was AT-rich in the three species. These results suggest high conservatism in the euchromatic regions as opposed to the heterochromatic regions that have a high rate of changes. In this study, we report the karyotype of *Trypoxylon rugifrons* F. Smith, 1873which has the lowest chromosome number in the genus and other characteristics of the likely ancestral *Trypoxylon* karyotype.

## Introduction

The Hymenoptera (Bees, wasps and ants) have received a remarkable amount of attention due to their amazing diversity of species, life histories, social behaviors, and key role in very diverse ecosystems. Their importance arises from their role as pollinators and biocontrol agents, their damage to agriculture and forestry, and their use as model organisms for the study of genetics and evolution (Ronquist 1999, Savard et al. 2006, Goodisman et al. 2008).

*Trypoxylon* Latreille, 1796 is a genus of solitary mud-dauber spider-hunting wasps that construct tubular nests entirely of mud or in preexisting tubular cavities. The genus comprises 660 species divided among two subgenera, *Trypoxylon* s.str. with a cosmopolitan distribution and *Trypargilum* Richards, 1934 restricted to the Western Hemisphere (Coville 1982, Hanson and Menke 1995).

Chromosome number and other karyological features are good sources of evidence for the systematics and species level taxonomy of several Hymenoptera groups. A marked chromosomal variability, even within species, has been reported for several aculeate Hymenoptera (Hoshiba et al. 1989). Developments in cytogenetic methods have opened the possibility of examining variation in chromosome number and structure in natural populations, therefore improving and expanding our knowledge of wasp karyology (Hoshiba et al. 1989, Gokhman and Quicke 1995, Gokhman 2010). However, cytogenetics has rarely been applied to the study of *Trypoxylon* and, so far, only 13 of the named 660 species have a known chromosome number.

In *Trypoxylon*, chromosome numbers range from 2n=18 to 2n=34 (Hoshiba and Imai 1993, Gomes et al. 1995, 1997) and two occurrences of intraspecific karyotype variation have been detected. In *Trypoxylon albitarse* Fabricius, 1804, karyotype variation was due to the presence of B chromosomes (Araújo et al. 2000). Scher and Pompolo (2003) also found remarkable karyotype differences (n=12 to 15 and 2n =25 to 28 and 2n=30) in a population of *Trypoxylon nitidum* F. Smith, 1856 in southeastern Brazil. Heterochromatin has been shown to be highly variable in the hymenopterans and has been specially meaningful in the evolutionof *Trypoxylon* karyotypes due to its high content in some species (Gomes et al. 1995, 1997, Scher and Pompolo 2003).

Previous cytogenetic analyses in *Trypoxylon* have been focused on C-banding, which by itself may not be sufficiently informative for a reliable heterochromatin description and comparative analysis of different species. Therefore, other techniques for molecular characterization can be very useful for this purpose. To improve qualitatively the data available so far, we applied in combination C-banding and base-specific fluorochrome staining to the chromosomes of three *Trypoxylon* species from the Atlantic rainforest in southern Bahia, in the Brazilian Northeast.

## Material and methods

Larvae of *Trypoxylon (Trypargilum) nitidum* (eight ♀ and three ♂), *Trypoxylon (Trypargilum) lactitarse* Saussure, 1867 (three ♀ and three ♂) and *Trypoxylon (Trypoxylon) rugifrons* F. Smith, 1873 (three ♀ and one ♂) were collected in the field directly in the wasp nests for cytogenetic analyses.

Specimens of *Trypoxylon nitidum* and *Trypoxylon lactitarse* were captured using trap-nests made of bamboo shoots sectioned below each node with 1 cm diameter or tubes of cardboard with one end closed with the same material and 0.7 cm diameter. The trap-nests were set up in Camacan (15°25'S, 39°29'W) and Ilhéus (14°47'S, 39°12'W), in the state of Bahia and were inspected twice a week. The traps containing complete nests were closed and taken to the Laboratório de Citogenética at the Universidade Estadual de Santa Cruz for the collections of specimens in the prepupal stage. Specimens of *Trypoxylon rugifrons* were captured from naturally occurring nests in Ilhéus.

At least two specimens per nest were kept at 28°C in a biochemical oxygen demand (BOD) incubator and daily monitored until adult emergence, at which time they were identified. Voucher specimens were deposited at the Entomological Collection at the Universidade Estadual de Santa Cruz.

Slides for cytogenetic analysis were prepared from cerebral ganglia of prepupae following Imai et al. (1988a). The slides containing metaphases were submitted to conventional Giemsa staining and C-banding (Sumner 1972, Pompolo and Takahashi 1990) to determine patterns of constitutive heterochromatin distribution. CMA3/DAPI double staining followed Schweizer (1980) with minor modifications by Guerra and Souza (2002).

At least 10 metaphases per specimen were observed using an epifluorescence microscope DMRA2 (Leica) and images were captured using the IM50 Leica software (Leica Microsystems Imaging Solutions Ltd, Cambridge, UK). Giemsa-stained chromosomes were photographed using an Olympus BX51 microscope equipped with an Olympus C-7070 digital camera. Digital images and figure mounting were prepared using Adobe Photoshop CS3 Extended version 10.0.

Chromosomes were described according to Imai’s terminology (Imai 1991) with acrocentric chromosome (A); pseudoacrocentric chromosome (AM); pseudoacrocentric chromosome with a heterochromatic block in the proximal region of the centromere of the euchromatic arm (AMC); metacentric heterochromatic chromosome (Mh); metacentric chromosome with an interstitial heterochromatic block in one of the arms (MC); metacentric chromosome with an interstitial heterochromatic block (MCC) (see the original reference for more details).

## Results

**Figure 1A–B.**
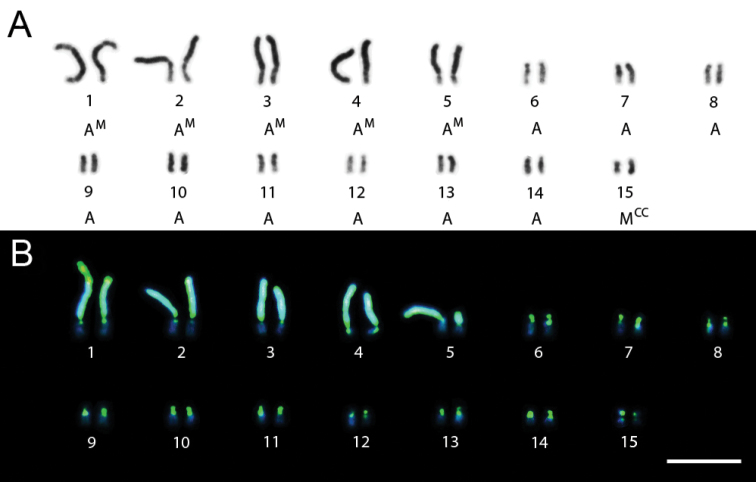
*Trypoxylon (Trypargilum) nitidum*. **A** Female karyotype (2n=30), standard staining **B** CMA3/DAPI staining of female karyotype (2n=30). Bar=10µm.

Specimens of *Trypoxylon nitidum* from Camacan had 2n=30 and n=15 chromosomes and the diploid karyotype formula was 2K=2MCC+10AM+18A (Figs 1A and 1B). Fluorochrome staining (Fig. 1B) revealed that pairs 1 to 5 (AM chromosomes) showed predominantly compacted heterochromatin (equally CMA3/DAPI stained) with balanced GC and AT composition over the long arm, except for the 1st pair, which showed a terminal euchromatic CMA3+/DAPI- (GC-rich) block. The heterochromatin of the acrocentric (6th to 14th pairs) and metacentric (15th pair) chromosomes showed a CMA3+/DAPI- pattern (Fig. 1B) whereas the remaining euchromatin in all chromosomes was CMA3-/DAPI+ (AT-rich). Karyotypes also showed size heteromorphism on the 1st and 5th pairs (Fig. 1B).

**Figure 2A–B.**
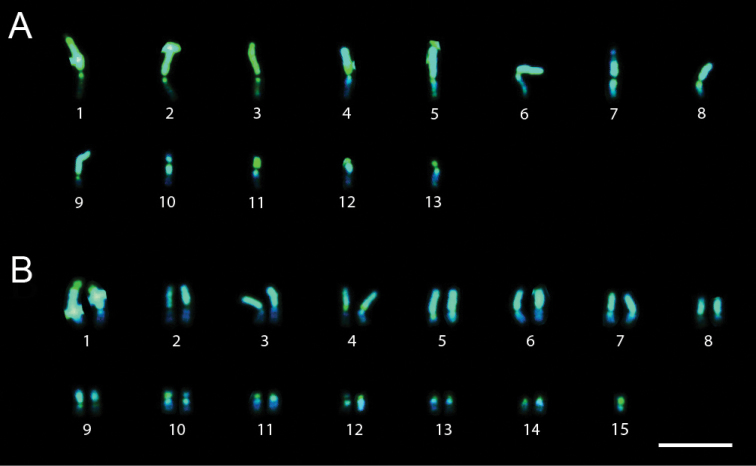
*Trypoxylon (Trypargilum) nitidum*. **A** Male karyotype (n=13) with CMA3/DAPI banding **B** CMA3/DAPI staining of female karyotype (2n=29). Bar=10µm.

Specimens of *Trypoxylon nitidum* collected in Ilhéus showed chromosome numbers 2n=29 and n=15 or n=13. The fluorochrome staining pattern also differed in these karyotypes. The 4th pair showed an interstitial CMA3+/DAPI- block near the centromeric region of one of the arms, chromosome pair 15 (Fig. 2B) had the long arm with a CMA3+/DAPI- pattern and chromosome pairs 11 and 13 (Fig. 2A) showed short arms with a CMA3+/DAPI- pattern and long arms with a CMA3-/DAPI+ pattern.

**Figure 3A–B.**
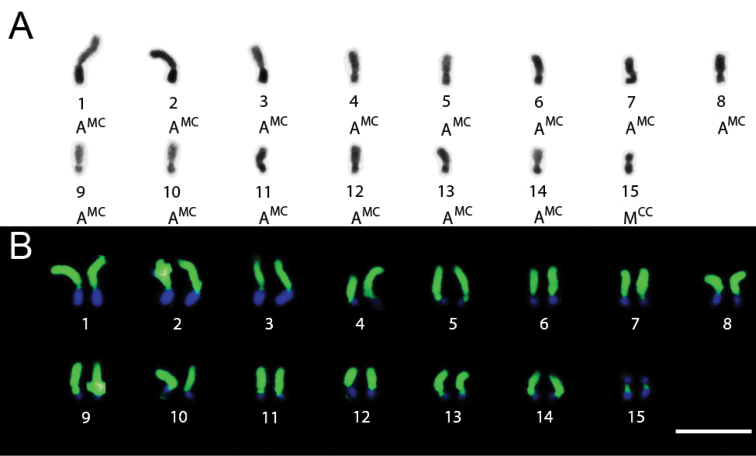
*Trypoxylon (Trypargilum) lactitarse*. **A** Male karyotype (n=15) with standard staining **B** CMA3/DAPI staining of female karyotype (2n=30). Bar=10µm.

*Trypoxylon lactitarse* had 2n=30 and n=15 chromosomes and the karyotype formula 2K = 2MCC +28AMC (Figs 3A and 3B). The 4th pair was heteromorphic with variation in the length of the long arm (Fig. 3B). The heterochromatin in all chromosomes showed a CMA3+/DAPI- pattern, whereas the euchromatin showed a CMA3-/DAPI+ pattern (Fig. 3B).

**Figure 4A–C.**
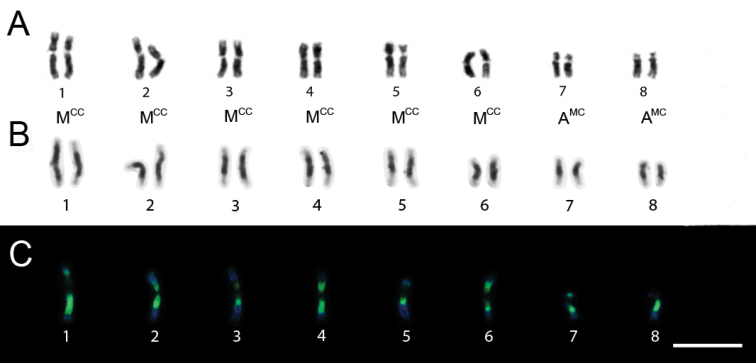
*Trypoxylon (Trypoxylon) rugifrons.* **A** Female karyotype (2n=16) with standard staining **B** C-banding patterns in female karyotype (2n=16) **C** CMA3/ DAPI staining of male karyotype (n=8). Bar=10µm.

*Trypoxylon rugifrons* had 2n=16 and n=8 chromosomes and the observed karyotype formula was 2K=12MCC+4AMC (Figs 4A and 4B). The heterochromatin in all chromosomes was CMA3+/DAPI- and the euchromatin showed a CMA3-/DAPI+ pattern (Fig. 4C).

## Discussion

A large variation in chromosome number has been reported for several Hymenoptera groups (Imai et al. 1977, Crosland and Crozier 1986, Pompolo and Takahashi 1987, 1990, Imai and Taylor 1989, Costa et al. 1993, Gokhman 2010). Specimens of *Trypoxylon nitidum* and *Trypoxylon lactitarse* showed a chromosome number within the range observed in this subgenus, whereas *Trypoxylon rugifrons* specimens showed the lowest chromosome number reported in the genus so far. The karyotypes and C-banding in *Trypoxylon lactitarse* and *Trypoxylon nitidum* from Camacanwere similar to those found by Gomes et al. (1997) and Scher and Pompolo (2003) in the Brazilian Southeast region, revealing karyotype stability within these species along this range of the geographic distribution.

Regarding chromosome morphology, *Trypoxylon rugifrons* showed predominantly metacentric and submetacentric chromosomes (pairs 1 to 6) and heterochromatin concentrated in the pericentromeric regions. These chromosomes are larger than those of the pairs 7 and 8. Gomes et al. (1997) proposed that the ancestral karyotype of *Trypoxylon* would probably have had n = 7 or 8 chromosomes and that an increase in chromosome number due to centric fissions led to the higher chromosome number registered thus far in the group. Their assumption was based on Imai et al.´s (1986, 1988a, b, 1994) minimum interaction hypothesis for karyotype evolution, which proposes that chromosome numbers tend to increase by centric fission and this process could be evolutionarily favored by the reduction of physical interaction between non-homologous chromosomes, therefore minimizing the genetic risks of deleterious translocations during meiosis.

The predominance of highly heterochromatic pseudoacrocentric chromosomes in most species within the genus *Trypoxylon* and the reduced chromosome number and chromosome morphology showed by *Trypoxylon rugifrons* lend support to Gomes et al.´s (1997) hypothesis. *Trypoxylon rugifrons* with n = 8 could show features similar to those of the putative ancestral karyotype.

The specimens of *Trypoxylon nitidum* from Ilhéus with 2n=29 also had a small metacentric chromosome (15th pair) with a GC-rich arm whose homologue was not present. These results have also revealed band similarities between the GC-rich arm and the terminal region of the 1st pair, which is also GC-rich (Fig. 2B). This evidence leads us to infer that a fusion between the 15th pair and the terminal region of one of the chromosomes of pair 1 could be involved in this numeric variation. This could also explain the heteromorphism of a CMA3+/DAPI- block present in one of the homologues of the 1st pair (Fig. 1B). Araújo et al. (2000) verified that the B chromosome in *Trypoxylon albitarse* was also stained by CMA3, similarly to the constitutive heterochromatin of all chromosomes in the complement, thus suggesting that the B chromosome originated from breaks in the constitutive heterochromatin and is maintained in the genome by centromeric “reactivation”. Scher and Pompolo (2003) did not report *Trypoxylon nitidum* specimens with 2n=29 in a population collected at the Parque Estadual do Rio Doce, in the state of Minas Gerais (19°30'S, 41°1'W). This heterogeneity in karyotype over the distribution range highlights the need for further investigations in different localities to better resolve both karyotype distribution mapping and variation among *Trypoxylon nitidum* populations. Further investigation will also be useful to confirm or reject whether the variable chromosomes are accessory in *Trypoxylon nitidum*.

The heteromorphism found in the 5th pair in *Trypoxylon nitidum* and in the 4th pair in *Trypoxylon lactitarse* could be explained by a deletion or, alternatively, by a tandem growth of the heterochromatic blocks in the long arms. A previous study by Gomes et al. (1997) also found heteromorphism in *Trypoxylon nitidum*, which those authors attributed to a deletion in one of the homologues. Based on the current knowledge on this matter, we consider both possibilities equally likely.

Several studies have demonstrated a correspondence between CMA3+ bands and rDNA sites in species such as *Scaptotrigona xanthotricha* Moure, 1950 and *Melipona* Illiger, 1806 bees (Rocha et al. 2002, Duarte et al. 2009), *Donax trunculus* L., 1758 (Mollusca: Bivalvia) (Petrović et al. 2009) and *Citrus* L., 1753 plants (Silva et al. 2010). It is therefore reasonable to predict that the CMA3+/DAPI- block in the terminal region of the 1st pair in all specimens of *Trypoxylon nitidum* may bear rDNA. The absence of a CMA3+/DAPI- block in *Trypoxylon lactitarse* and *Trypoxylon rugifrons* in euchromatic regions may be attributed to differences in the molecular composition of the rDNA. Using fluorescent *in situ* hybridization with an rDNA probe in *Trypoxylon albitarse*, Araújo et al. (2002) found that all rRNA genes are located on the heterochromatic arm in a single chromosome pair.

The three species studied herein are similar in their heterochromatin composition. However, the heterochromatin of the pseudoacrocentric chromosomes in *Trypoxylon nitidum* has a balanced GC and AT composition in contrast with the acrocentric and metacentric chromosomes, which heterochromatin is GC-rich. This result indicates a difference in the heterochromatin composition in *Trypoxylon nitidum*. Intraspecific variation in heterochromatin base composition has already been detected in other studies. For example, Domingues et al. (2005) detected a single chromosome pair with GC-rich heterochromatin in contrast with all the remaining AT-rich chromosomes in *Trigona fulviventris* Guérin, 1835 using C-banding and fluorochrome staining. Together with the previously described karyotypes, our results document a high frequency of large pseudoacrocentric chromosomes in Neotropical *Trypoxylon* species. These results lead us to believe that, regardless of base composition of the heterochromatin, the process of heterochromatin amplification has played an important role in the karyotype evolution of Neotropical fauna of aculeate Hymenoptera.

The euchromatin was AT-rich in the three species studied, which agrees with Araújo et al. (2000) who also found AT-rich euchromatin in *Trypoxylon albitarse*. Our results indicate a high conservatism of the euchromatic regions as opposed to the heterochromatic regions that have a high rate of change. Repetitive segments such as the heterochromatin are considered hot spots of chromosomal rearrangements (Eichler and Sankoff 2003). Due to the high heterochromatin content, it is particularly likely that these species are undergoing a process of rapid genome reorganization that manifests itself through the chromosome variation and heteromorphisms found in this study. Our results, as well as previously published studies show a high karyotype variation in *Trypoxylon*. However, the genus is still poorly known and lacks a taxonomic reevaluation under a phylogenetic approach that could allow robust assumptions to be drawn on the pattern of its chromosome evolution.

## References

[B1] Araújo SMSR Pompolo SG Dergam JAS Campos LAO (2000) The B chromosome system of *Trypoxylon* (*Trypargilum*) *albitarse* (Hymenoptera, Sphecidae) I. Banding analysis. Cytobios 101:7-13 10697741

[B2] Araújo SMR Pompolo SG Perfectti F Camacho JPM (2002) Genetic load caused by variation in the amount of rDNA in a wasp. Chromosome Research 10:607-613 doi: 10.1023/A:1020970820513 12498349 10.1023/a:1020970820513

[B3] Costa MA Melo GAR Pompolo SG Campos LAO (1993) Karyotypes and heterochromatin distribution (C-band patterns) in three species of *Microstigmus* wasps (Hymenoptera, Sphecidae, Pemphredoninae). Brazilian Journal of Genetics 16:923-926

[B4] Coville RE (1982) Wasps of the genus *Trypoxylon* subgenus *Trypargilum* in North America. University of California Press, Berkeley, 147 pp.

[B5] Crosland MWJ Crozier RH (1986) *Myrmecia pilosula*, an ant with only one pair of chromosomes. Science 231: 1278 doi: 10.1126/science.231.4743.1278 10.1126/science.231.4743.1278 17839565

[B6] Domingues AMT Waldschmidt AM Andrade-Souza V Silva JJC Costa MA (2005) Karyotype characterization of *Trigona fulviventris*, Guérin, 1835 (Hymenoptera, Meliponini) by C banding and fluorochrome staining: report of a new chromosome number in the genus. Genetics and Molecular Biology 28:390-393 doi: 10.1590/S1415-47572005000300009

[B7] Duarte OM Martins CC Waldschmidt AM Costa MA (2009) Occurrence of multiple nucleolus organizer regions and intraspecific karyotype variation in *Scaptotrigona xanthotricha* Moure (Hymenoptera, Meliponini). Genetics and Molecular Biology 8 (3):831-839 10.4238/vol8-3gmr598 19731205

[B8] Eichler EE Sankoff D (2003) Structural dynamics of eukaryotic chromosome evolution. Science 301:793-797 doi: 10.1126/science.1086132 12907789 10.1126/science.1086132

[B9] Gokhman VE Quicke DLJ (1995) The last twenty years of parasitic Hymenoptera karyology: an update and phylogenetic implications. Journal of Hymenoptera Research 4:41-63

[B10] Gokhman VE (2010) Chromosomes of parasitic wasps of the genus *Metaphycus* (Hymenoptera: Chalcidoidea: Encyrtidae). Comparative Cytogenetics 4 (1):21-25 doi: 10.3897/compcytogen.v4i1.29 10.3897/CompCytogen.v14i3.56535 PMC9849058 36761105

[B11] Gomes LF Pompolo SG Campos LAO (1995) Cytogenetic analysis of three species of *Trypoxylon* (*Trypoxylon*) (Hymenoptera, Sphecidae, Larrinae). Brazilian Journal of Genetics 18:173-176

[B12] Gomes LF Pompolo SG Campos LAO (1997) Karyotype evolution in wasps of the genus *Trypoxylon* (subgenus *Trypargilum*) (Hymenoptera, Sphecidae). Brazilian Journal of Genetics 20:177-183

[B13] Goodisman MAD Kovacs JL Hunt BG (2008) Functional genetics and genomics in ants (Hymenoptera: Formicidae): The interplay of genes and social life. Myrmecological news 11:107-117

[B14] Guerra M Souza MJ (2002) Como Observar Cromossomos: Um Guia de Técnicas em Citogenética Vegetal Animal e Humana. FUNPEC Editora. 13 2 pp.

[B15] Hanson PE Menke AS (1995) The sphecid wasps (Sphecidae). In: Hanson PE Gauld ID (Eds) The Hymenoptera of Costa Rica. Oxford University Press, New York, 621–649

[B16] Hoshiba H Matsuura M Imai HT (1989) Karyotype evolution in the social wasps (Hymenoptera, Vespidae). Japanese Journal of Genetics 64:209-222 doi: 10.1266/jjg.64.209

[B17] Hoshiba H Imai HT (1993) Chromosome evolution of bees and wasps (Hymenoptera, Apocrita) on the basis of C-banding pattern analyses. Japanese Journal of Genetics 61 (3):465-492

[B18] Imai HT Crozier RH Taylor RW (1977) Karyotype evolution in Australian ants. Chromosoma 53:341-393 doi: 10.1007/BF00327974

[B19] Imai HT Maruyama T Gojobori T Inoue Y Crozier RS (1986) Theoretical bases for karyotype evolution. 1. The minimum-interaction hypothesis. The American Naturalist 128:900-920 doi: 10.1086/284612

[B20] Imai HT Taylor RW Crosland MWJ Crozier RH (1988a) Modes of spontaneous chromosomal mutation and karyotype evolution in ants with reference to the minimum interaction hypothesis. Japanese Journal of Genetics 63:159-185 doi: 10.1266/jjg.63.159 3273765 10.1266/jjg.63.159

[B21] Imai HT Takahata N Maruyama T Daniel A Honda T Matsuda Y Moriwaki K (1988b) Theoretical bases for karyotype evolution. II. The fusion burst in man and mouse. Japanese Journal of Genetics 63:313-342 doi: 10.1266/jjg.63.313 3078875 10.1266/jjg.63.313

[B22] Imai HT Taylor RW (1989) Chromosomal polymorphisms involving telomere fusion, centromeric inactivation and centromere shift in the ant *Myrmecia* (*pilosula*) *n = 1*. Chromosoma 98:456-460 doi: 10.1007/BF00292792

[B23] Imai HT (1991) Mutability of constitutive heterocromatin (C-bands) during eukaryotic chromossomal evolution and their cytological meaning. Japanese Journal of Genetics 66:653-661 doi: 10.1266/jjg.66.635 10.1266/jjg.66.635 1777253

[B24] Imai HT Taylor RW Crozier RH (1994) Experimental bases for the minimum interaction theory. I. Chromosome evolution in ants of the *Myrmecia pilosula* species complex (Hymenoptera: Formicidae: Myrmeciinae). Japanese Journal of Genetics 69:137-182 doi: doi:10.1266/jjg.69.137

[B25] Petrović V Pérez-García C Pasantes JJ Šatović E Prats E Plohl M (2009) A GC-rich satellite DNA and karyology of the bivalve mollusk *Donax trunculus*: a dominance of GC-rich heterochromatin. Cytogenetic and Genome Research 124:63-71 doi: 10.1159/000200089 19372670 10.1159/000200089

[B26] Pompolo SG Takahashi CS (1987) Cytogenetics of Brazilian Polybiini wasps (Hymenoptera, Vespidae, Polistinae). Brazilian Journal of Genetics 10:483-496

[B27] Pompolo SG Takahashi CS (1990) Chromosome numbers and C-banding in two wasp species of the genus *Polistes* (Hymenoptera, Polistinae, Polistini). Insectes Sociaux 37:251-257 doi: 10.1007/BF02224052

[B28] Rocha MP Pompolo SG Dergam JA Fernandes A Campos LAO (2002) DNA characterization and karyotypic evolution in the bee genus *Melipona* (Hymenoptera, Meliponini). Hereditas 136:19-27 doi: 10.1034/j.1601-5223.2002.1360104.x 12184485 10.1034/j.1601-5223.2002.1360104.x

[B29] Ronquist F (1999) Phylogeny of the Hymenoptera (Insecta): The state of the art. Zoologica Scripta 28 (1–2):3-11 doi: 10.1046/j.1463-6409.1999.00019.x

[B30] Savard J Tautz D Richards S Weinstock GM Gibbs RA Werren JH Tettelin H Lercher MJ (2006) Phylogenomic analysis reveals bees and wasps (Hymenoptera) at the base of the radiation of Holometabolous insects. Genome Research 16:1334-1338 doi: 10.1101/gr.5204306 17065606 10.1101/gr.5204306 PMC1626634

[B31] Scher R Pompolo SG (2003) Evolutionary dynamics of the karyotype of the wasp *Trypoxylon* (*Trypargilum*) *nitidum* (Hymenoptera, Sphecidae) from the Rio Doce state Park, Minas Gerais, Brazil. Genetics and Molecular Biology 26:307-311 doi:10.1590/S1415-47572003000300015

[B32] Schweizer D (1980) Simultaneous fluorescent staining of R bands and specific heterochromatic regions (DA-DAPI bands) in human chromosomes. Cytogenetics and Cell Genetics 27:190-193 doi: 10.1159/000131482 6156801 10.1159/000131482

[B33] Silva AEB Marques A Santos KGB Guerra M (2010) The evolution of CMA bands in *Citrus* and related genera. Chromosome Research 18:503-514 doi: 10.1007/s10577-010-9130-2 20490650 10.1007/s10577-010-9130-2

[B34] Sumner AT (1972) A simple technique for demonstration of centromeric heterochromatin. Experimental Cell Research 75:304-306 doi: 10.1016/0014-4827(72)90558-7 4117921 10.1016/0014-4827(72)90558-7

